# Gut Microbiota Affects Host Fitness of Fall Armyworm Feeding on Different Food Types

**DOI:** 10.3390/insects15050304

**Published:** 2024-04-24

**Authors:** Lin Ma, Daotong Wang, Qilin Ren, Jiaqi Sun, Lei Zhang, Yunxia Cheng, Xingfu Jiang

**Affiliations:** State Key Laboratory for Biology of Plant Diseases and Insect Pests, Institute of Plant Protection, Chinese Academy of Agricultural Science, Beijing 100193, China; linma1990@163.com (L.M.); wang_dt97@163.com (D.W.); qlren94@163.com (Q.R.); sjq031015@163.com (J.S.); leizhang@ippcaas.cn (L.Z.); yxcheng@ippcaas.cn (Y.C.)

**Keywords:** *Spodoptera frugiperda*, host adaptation, gut microbiota, life history, nutrient utilization

## Abstract

**Simple Summary:**

The fall armyworm, a migratory invasive pest, poses a serious threat to the food security of major crops like maize, wheat, and rice. Understanding its adaptation mechanism to different hosts is crucial for developing effective control technology. This study examined the host fitness and gut microbial diversity of fall armyworms fed four different types of food. Based on life history parameters, pupa weight, and nutrient utilization indexes, the host fitness ranking from high to low was artificial diet, maize, wheat, and rice. Gut microbial composition and diversity varied significantly among fall armyworms fed different foods due to changes in low-abundant bacteria. Fall armyworms fed maize had the highest gut microbial diversity. The functions of gut microbes with significant abundance differences were enriched in nutrient and vitamin metabolism as well as other pathways closely related to host adaptation. Additionally, we identified five genera (*Acinetobacter*, *Variovorax*, *Pseudomonas*, *Bacillus*, and *Serratia*) that positively correlated with host fitness, while one genus (*Rahnella*) negatively correlated with it. This study reveals the potential role of gut microbes in the host adaptation of fall armyworms.

**Abstract:**

The fall armyworm (FAW), *Spodoptera frugiperda*, seriously threatens food and cash crops. Maize, wheat, and even rice damage by FAWs have been reported in many areas of China. It is urgent to clarify the mechanism which FAWs adapt to different feeding hosts and develop effective control technologies. Two-sex life tables and 16s rDNA sequencing were used to determine the host fitness and gut microbial diversity of FAWs when fed four different food types. Considering the life history parameters, pupa weight, and nutrient utilization indexes, the host fitness of FAWs when fed different food types changed in descending order as follows: artificial diet, maize, wheat, and rice. The gut microbial composition and the diversity of FAWs when fed different food types were significantly different, and those changes were driven by low-abundant bacteria. The gut microbes of FAWs that were fed with maize had the highest diversity. The functions of the gut microbes with significant abundance differences were enriched in nutrient and vitamin metabolism and other pathways that were closely related to host adaptation. Furthermore, we identified five genera (*Acinetobacter*, *Variovorax*, *Pseudomonas*, *Bacillus*, and *Serratia*) and one genus (*Rahnella*) that were positively and negatively correlated with the host fitness, respectively. This study revealed the possible role of gut microbes in the host adaptation of FAWs.

## 1. Introduction

Due to its wide host range, strong migratory ability, and strong species competitiveness, the fall armyworm (FAW), *Spodoptera frugiperda* (Smith), seriously threatens food and cash crops, including maize, wheat, and cotton [1,2,3]. Meanwhile, being a major migratory pest, the speed at which this pest invades and spreads is concerning. After infesting Myanmar in late 2018, FAW spread to 27 Chinese provinces within a year and became a dominant pest [4,5].

Researchers have carried out extensive research on the host adaptation of/fitness differences in FAWs in different host plants [6,7]. In general, the FAWs invading China exhibit the highest host adaptability to maize [8,9]. In addition to maize, wheat has become an important wintering host plant of FAWs [10,11]. Furthermore, there have been reports of FAW infestations in rice fields across many areas of China [12,13,14]. The *COI*-R *Tpi*-C configuration, detected using the *COI* and *Tpi* (MspI) gene combination identification method, represents the primary host biotype that initially arrived in China [15,16]. Due to these reasons, the potential for FAWs to harm food crops other than maize must be considered. Therefore, it is crucial to investigate the adaptation mechanisms of FAWs to different feeding hosts such as maize, wheat, and rice. 

In addition to studying the effects of different feeding hosts on insect life history parameters, the consumption and utilization of insects on different feeding hosts should not be ignored in the process of studying the formation mechanism of insect host adaptation [17,18]. When examining the food consumption, utilization, and detoxication enzyme (MFO: mixed-function oxidase) activities of two FAW host strains that fed on maize and rice, the results indicated that both behavioral and physiological factors were associated with host use [19]. The metabolization of foreign chemicals is among the key functions involved in the phenotypic variation in FAW strains [20]. Hafeez et al. [21] indicates the possible roles of *S. frugiperda* gut digestive protease enzymes and related genes (*SfTry-3*, *SfTry-7*, and *Sfchym-9*) in host plant adaptation. The consumption and use of food by insects not only represent their acceptance but also provide basic conditions for their growth, development, and reproduction, which may be the key factor affecting the formation of insect host adaptation characteristics [22,23].

Gut microbiotas play an important role in plant–insect interactions [24], including insect feeding and host digestion, insect–plant defense and immune resistance, and insect growth and development [25,26,27]. With the introduction of the holo-genome evolutionary theory and the development of high-throughput sequencing [28], more attention has been paid to the role of gut microbes in host adaptability [29,30,31]. There is growing evidence that variation in gut microbes can influence insect host phenotypes [32,33,34]. It has been proved that the *Pseudomonas* strain in the gut enables *Hypothenemus hampei* to acquire a caffeine degradation ability, allowing *H. hampei* to better utilize coffee beans [35]. Although some studies on the gut bacteria of FAWs have been conducted [36], it remains unclear whether the gut microbiotas of FAWs play a role in their adaptation to various host plants.

This research was devoted to investigating the potential role of the gut bacteria of FAWs in adapting to different host plants. In this study, four different food types were used, including an artificial diet and three host plants. The host fitness (life table parameters, pupal weight, and nutrient utilization indexes) of FAWs when feeding on different food types were measured. The gut bacterial compositions of FAWs feeding on different feed types were determined by 16s rDNA sequencing to explore the taxonomic diversity in different groups. By conducting correlation analysis between host fitness and gut bacterial taxa, we aimed to gain a better understanding of the interaction between microbes and insect host adaptations. Our research aimed to uncover the possible role of FAWs’ gut microbiotas in strategies that enable their adaptation to various host plants.

## 2. Materials and Methods

### 2.1. Insects and Food Types

FAWs were collected from maize fields in Nanning city, Guangxi Zhuang Autonomous Region (108.37° E, 22.82° N), in April 2019, and subsequently fed an artificial diet (for details, see Table S1) to establish a population for multiple generations under laboratory conditions. To ensure a consistent genetic background, we selected eggs laid after the mating of one adult male and one adult female from the laboratory population for subsequent experiments. After enough eggs were produced, we used the *COI* and *Tpi* [MspI] gene combination identification method [37] to determine the biotype of the parents, which both had the *COI*-R *Tpi*-C configuration. The environmental conditions in the incubator for raising FAWs were set at 27 ± 1 °C, with a light–dark cycle of 14:10 h and a relative humidity of 60–80%. Adults were fed with a 10% (*w*/*v*) honey–water mixture.

The artificial diet and three host plants were used as four different food types. The host plants, maize (*Zea mays* L.; Zhengdan 958, provided by the Institute of Crop Sciences, the Chinese Academy of Agricultural Sciences), wheat (*Triticum aestivum* L.; Luyuan 502, provided by the Institute of Crop Sciences, the Chinese Academy of Agricultural Sciences), and rice (*Oryza sativa* L.; Zhenghan 10, provided by the Henan Academy of Agricultural Sciences), were cultured in a greenhouse at 25 ± 1 °C at the Institute of Plant Protection, the Chinese Academy of Agricultural Sciences, Beijing, China. The host plants were fed to FAWs at the seedling stage (2–3 weeks after emergence).

### 2.2. Performance of FAWs Feeding on Different Food Types

Differences in the performance of FAWs when feeding on different food types were assessed by measuring the life table parameters, pupal weight, and nutrient utilization indexes. 

According to Chi’s study [38], two-sex life tables were established for FAWs fed on four food types, respectively. For each food type, one hundred newly laid eggs from mated females were individually placed into 6-well plates and covered with toilet paper and a lid to prevent escape. Each FAW from the 1st to the 6th instar larva and the pupal stage was kept in the 6-well plate with food. The survival and development times of FAWs in each stage were recorded daily. The newly emerged females were individually paired with young males from the colony in a glass tube (5 × 12 cm diameter × height) and covered with cotton gauze as the oviposition substrate. The pairs were fed 10% (*w*/*v*) honey in sterile water. The survival and the number of eggs laid were recorded daily until all FAW adults died. The raw life history data were analyzed based on an age-stage, two-sex life table using TWO-SEX-MSChart software (v2023) [39]. The performances (developmental time of each stage; *R*_0_, net reproduction; *r*, intrinsic rate of increase; *λ*, finite rate; T, mean generation time; APOP, adult preoviposition period; oviposition days; and mean fecundity) of the FAW groups were calculated using TWO-SEX-MSChart (v2023).

Following the life table establishment, the 4th-day pupal weight of thirty FAWs was measured.

Twenty 6th-1st day FAW larvae that fed on each food type were used to determine the nutrient utilization indexes, which were measured as described in [22]. After a 6 h starvation treatment, each larva continued to feed on the corresponding food type for 24 h. The fresh weight and dry/fresh weight ratio of the larvae before feeding, the fresh weight and dry/fresh weight ratio of the food before feeding, the fresh weight and the dry weight of larvae after feeding for 24 h, including the dry weight of uneaten food and the dry weight of feces, were determined. The calculated indexes included the relative growth rate (RGR), approximate digestibility (AD), relative consumption rate (RCR), the efficiency of conversion for digested food (ECD), and ingested food (ECI). The calculation formula for each index was as follows:

$$RCR=\frac{W_{FI}-W_{UF}}{\left[\left(W_{IL}+W_{FL}\right)/2\right]\times T}\times 100$$

$$RGR=\frac{W_{FL}-W_{IL}}{\left[\left(W_{IL}+W_{FL}\right)/2\right]\times T}\times 100$$

$$AD=\frac{W_{FI}-W_{UF}-W_{F}}{W_{FI}-W_{UF}}\times 100$$

$$ECD=\frac{W_{FL}-W_{IL}}{W_{FI}-W_{UF}-W_{F}}\times 100$$

$$ECI=\frac{W_{FL}-W_{IL}}{W_{FI}-W_{UF}}\times 100$$

*W_FI_*: dry weight of food introduced; *W_UF_*: dry weight of uneaten food; *W_IL_:* the initial dry weight of the larvae; *W_FL_*: the final dry weight of the larvae; *W_F_*: dry weight of feces.

### 2.3. Determining the Diversity of FAW Gut Microbial Communities after Feeding on Different Food Types

To clarify the differences in the gut microbiota diversities of FAWs induced by feeding on different food types, 16S rDNA sequencing was performed on the guts of 6th–1st day FAW larvae with the same genetic background that were continuously fed on different food types for more than five generations.

#### 2.3.1. Sample Collection and DNA Extraction

Four groups of FAW samples were collected and named according to the food types; the groups were as follows: artificial diet, maize, wheat, and rice. For each group, the guts of four 6th-1st day larvae were dissected and placed in a 2 mL sterile tube, with five replicates per group. DNA was extracted using the E.Z.N.A. ^®^Stool DNA Kit (D4015, Omega, Inc., Norwalk, CT, USA), according to the manufacturer’s recommendations. Nuclease-free water was used as a blank. The total DNA was eluted into 50 μL of elution buffer and stored at −80 °C until PCR amplification by LC-BioTechnology Co., Ltd. (Hangzhou, China).

#### 2.3.2. PCR Amplification and 16S rDNA Sequencing

The V3-V4 region of the 16S rRNA gene was amplified using the universal primers 341F (5′-CCTACGGGNGGCWGCAG-3′) and 806R (5′-GACTACHVGGGTATCTAATCC-3′). PCR amplification was performed in a reaction volume of 25 μL containing 25 ng template DNA, 12.5 μL PCR Premix, 2.5 μL of each primer, and PCR-grade water to adjust the volume. The PCR conditions included an initial denaturation at 98 °C for the 30 s, 32 denaturation cycles at 98 °C for the 10 s, annealing at 54 °C for the 30 s, an extension at 72 °C for 45 s, and the final extension at 72 °C for 10 min. The PCR products were confirmed with 2% agarose gel electrophoresis. Throughout DNA extraction, ultrapure water was used as a negative control to rule out false positives. The PCR products were purified using AMPure XT beads (Beckman Coulter Genomics, Danvers, MA, USA) and quantified by Qubit (Invitrogen, Carlsbad, CA, USA). The amplicon pools were prepared for sequencing, and the amplicon library size and quantity were assessed using the Agilent 2100 Bioanalyzer (Agilent, Santa Clara, CA, USA) and the Illumina Library Quantification Kit (Kapa Biosciences, Wilmington, MA, USA), respectively. The libraries were sequenced on the NovaSeq PE250 platform. The raw sequence reads are in the NCBI SRA database under the Project Accession ID PRJNA939966.

### 2.4. Data Analysis

The standard errors for each performance and significant differences among FAWs that fed on different food types were estimated using the paired bootstrap test in TWO-SEX-MSChart (v2023). Pupal weight and nutrient utilization indexes were compared using ANOVA, followed by Tukey’s HSD post hoc comparisons in SPSS 25.

For the 16S rDNA sequencing of the four groups of FAW gut microbial communities, paired-end reads were assigned to sequencing data samples based on their unique barcode and were truncated by removing the barcode and primer sequence. Paired-end reads were merged using FLASH. Raw and quality filtering was performed to obtain high-quality clean tags, according to fqtrim (v0.94). Chimeric sequences were filtered using Vsearch (v2.3.4). After dereplication using DADA2 [40], we obtained feature tables and sequences. Alpha and beta diversities were calculated by randomly normalizing them to the same sequences. Then, according to the SILVA (release 132, https://www.arb-silva.de/documentation/release-132/ (accessed on 24 October 2022) classifier, feature abundance was normalized using the relative abundance of each sample. The species diversity of each sample was analyzed using alpha diversity, Chao1, Observed_species, Goods_coverage, Shannon, and Simpson; these indices and the beta diversity were calculated using QIIME2 [41], and all graphs and diagrams were drawn in R (v3.5.2). Blast was used for sequence alignment, and feature sequences were annotated using the SILVA database (release 132). The PICRUSt2 (v2.2.0-b) [42] software (https://github.com/picrust/picrust2 (accessed on 24 October 2022) was used to predict the function of FAWs’ gut microbiota.

Spearman’s correlation was used to identify correlations between the biomarkers, which were selected from different groups of FAW gut microbial taxa, and FAW performance in R (v3.5.2), including the pupal weight (mg) and five nutrient utilization indexes. The relative abundance of gut bacterial taxa and FAW performance were $\log_{10}\left(raw\;data+1\right)$ transformed.

## 3. Results

### 3.1. Performances of FAWs Feeding on Different Food Types

The development and reproduction of the FAWs feeding on different food types showed significant differences. Compared with other food types, FAWs were fed an artificial diet, which showed the highest *R*_0_ (*p* < 0.05) (Table 1). FAWs feeding on rice had the lowest *r* and λ (*p* < 0.05) (Table 1). In comparison to those feeding on maize and wheat, FAWs that were fed the artificial diet and rice had a significantly longer T by approximately 2 days (*p* < 0.05) (Table 1). Different food types had little effect on the oviposition days of FAWs (*p* > 0.05), but the mean fecundity of reproductive females of FAWs when feeding on the artificial diet and maize was significantly higher than that of wheat and rice (*p* < 0.05) (Table 1).

**Figure 1:**
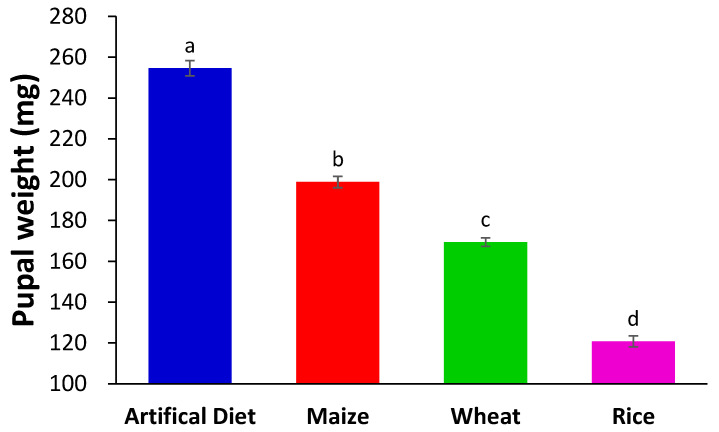
Pupal weight of FAWs feeding on different food types. Lowercase letters indicate the significance of the difference (compared using ANOVA, followed by Tukey’s HSD post hoc comparisons); the error bars indicate the standard errors.

The pupal weight of FAWs feeding on different food types was significantly different as follows: artificial diet > maize > wheat > rice (*F*_(3,119)_ = 372.45, *p* < 0.01) (Figure 1).

The water content of different food types varied significantly. Based on the dry/fresh weight ratio, maize had the highest water content, followed by rice and wheat, while the artificial diet had the lowest (*F*_(3,79)_ = 2001.40, *p* < 0.01) (Table 2). Therefore, dry weight was used to calculate nutrient use indicators. For each food type, when the 6th–1st day FAWs fed for 24 h, both the fresh weight and dry weight of worm bodies were heaviest with the artificial diet, followed by maize and wheat, and rice had the lightest weight (fresh weight: *F*_(3,79)_ = 36.17, *p* < 0.01; dry weight: *F*_(3,79)_ = 105.39, *p* < 0.01) (Table 2). FAWs exhibited significant differences in their ability to utilize and digest different food types. The RCR of FAWs when feeding on various food types showed the following pattern: maize > wheat ≈ rice > artificial diet (*F*_(3,79)_ = 101.27, *p* < 0.01) (Table 2). In other words, FAWs had the largest relative feed intake and the highest palatability of maize. The RGR of FAWs when feeding on various food types showed the following pattern: maize ≈ artificial diet > wheat > rice (*F*_(3,79)_ = 118.95, *p* < 0.01) (Table 2). In other words, the FAWs had the best absorption of maize during the artificial diet, followed by wheat, and the worst absorption was rice. The AD of FAWs when feeding on various food types showed the following pattern: artificial diet > maize > wheat > rice (F_(3,79)_ = 275.06, *p* < 0.01) (Table 2). In other words, FAWs digested the artificial diet better, followed by maize and wheat, with the least digestible being rice. The ECI of FAWs when feeding on various food types showed the following pattern: artificial diet > maize ≈ wheat > rice (F_(3,79)_ = 244.65, *p* < 0.01) (Table 2).

### 3.2. Diversity of FAW Gut Microbial Communities after Feeding on Different Food Types

After performing paired-end assembly, quality control, and chimera filtering on the raw reads, high-quality clean tags were obtained. The detailed statistical information of the raw and valid data for each sample can be found in Table S2. Based on the abundant table of features obtained after removing background noise using QIIME2, the number of common and unique features in each group was calculated. In total, 14, 152, 74, and 58 features were found in the four groups: artificial diet, maize, wheat, and rice, respectively. Only three features were shared among all groups, and 6, 128, 44, and 30 unique features were found in the four groups: artificial diet, maize, wheat, and rice, respectively (Figure S1). 

The α-diversity of the four group samples at the feature level was estimated using the Chao1, good-coverage, Shannon, and Simpson indices. There was no difference in the good-coverage index among all groups, which were close to one. This indicates that the probability of new species not being detected in the samples was low and suggests that the sequencing results likely represent the real situation (Wilcoxon test, *p* < 0.01; Figure S2B). The Chao1, Shannon, and Simpson indices showed significant differences among the four groups (Wilcoxon test for Chao1, *p* < 0.01; Wilcoxon test for Shannon, *p* < 0.01; Wilcoxon test, *p* < 0.01; Figure S2A,C,D). The α-diversity suggests that species richness and community microorganism diversity in gut of FAW larvae were highest in the maize group and lowest in the artificial diet group.

**Figure 2:**
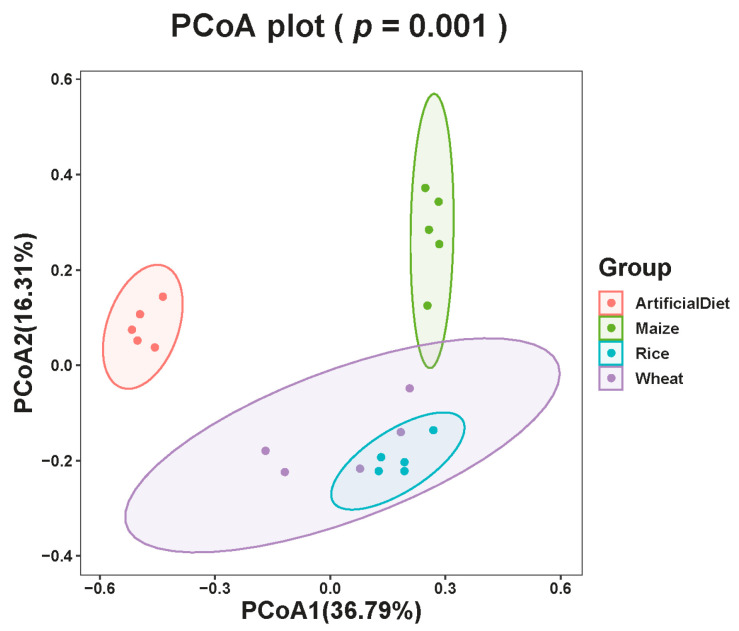
PCoA of gut bacteria in FAW fed different food types based on Bray–Curtis distances generated from feature tables. Different colors represent different groupings (adonis).

The β-diversity of four group samples at the feature level illustrated the similarities and differences in species composition and community structure. According to the principal coordinate analysis (PCoA), microbial communities were clearly divided into the following groups based on food types: artificial diet, maize, wheat, and rice (adonis, F_(3,19)_ = 7.06, R^2^ = 0.57, *p* < 0.01; Figure 2). ANOSIM analysis also shows that feeding different food types significant impact FAWs’ gut bacterial composition (R^2^ = 0.70, *p* < 0.01).

**Figure 3:**
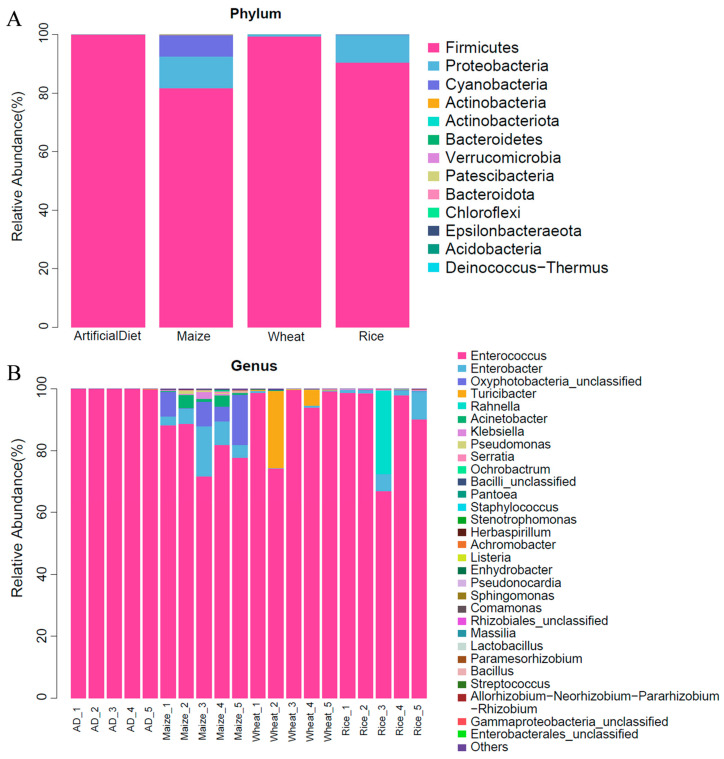
Relative abundance of gut microbiota phyla (**A**) and genera (**B**) of FAW larvae when fed different food types. Different colors represent the relative abundance of gut microbiota. ‘AD’ stands for artificial diet.

In total, 13 phyla, 20 classes, 43 orders, 79 families, and 128 genera were detected in the 244 features of all groups. The community composition was analyzed at the phylum and genus levels (Figure 3). At the phylum level, Firmicutes occupied a dominant position in all groups, especially in the group’s artificial diet and wheat, with an average relative abundance of 99.99 and 99.36%, and 81.74 and 90.53% for groups maize and rice, respectively. Proteobacteria and Cyanobacteria were also the most abundant phyla in the maize group, with an average relative abundance of 10.78 and 7.43%, respectively. Moreover, Proteobacteria was also the most abundant phyla in the rice group, with an average relative abundance of 9.36% (Figure 3A). At the genus level, FAW gut bacterial genera demonstrated different distributions depending on their food types. *Enterococcus* was a dominant genus in all groups, with an average abundance of more than 81.66%, especially in the artificial diet group, where the average abundance reached 99.98%. In addition to *Enterococcus*, the top genera with an average abundance of >1% in the maize group were *Oxyphotobacteria_unclassified*, *Enterobacter*, and *Acinetobacter*, while those in group wheat were *Turicibacter*, and those in the rice group were *Enterobacter* and *Rahnella* (Figure 3B). Samples from the maize group showed a more abundant gut microbiota diversity.

**Figure 4:**
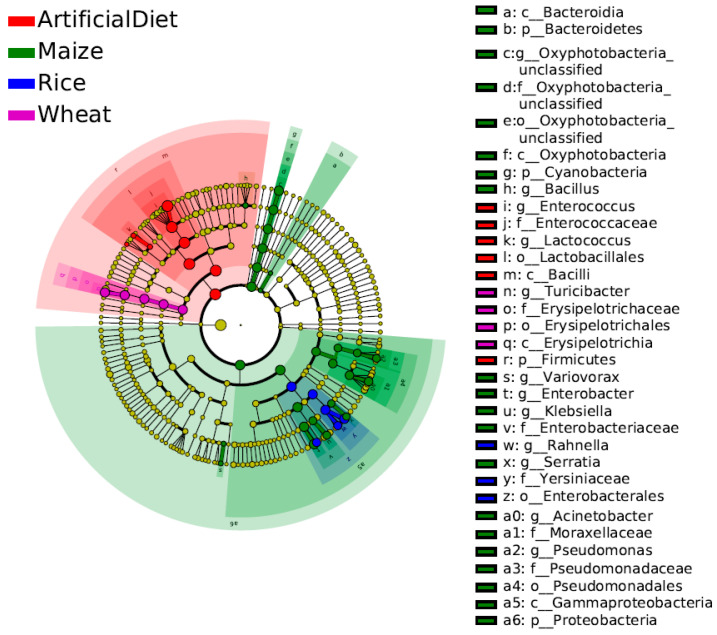
LEfSe analysis of gut microbiota in FAW larvae when fed different food types (LDA scores > 3). Different colors represent different groups; the phyla with significant differences are directly marked in the figure, and the significant differences in other levels are identified by letters.

LEfSe (LDA Effect Size) analysis was performed to further explore biomarkers in each group with significant differences in the relative abundance across other groups (Figure 4; LDA scores > 3). At the genus level, *Enterococcus* and *Lactococcus* were identified as biomarkers with a higher abundance in the artificial diet group. *Oxyphotobacteria*_unclassified, *Enterobacter*, *Acinetobacter*, *Variovorax*, *Klebsiella*, *Pseudomonas*, *Bacillus*, and *Serratia* were found as biomarkers with a higher abundance in the Maize group. Only one biomarker was found with a higher abundance in the Wheat and Rice groups respectively, which was *Turicibacter* and *Rahnella*.

Based on PICRUSt2 functional prediction, at KEGG Level 2, the results showed that the functions of gut microbiota with significant differences in abundance across the groups were mainly involved in glycan biosynthesis and metabolism, carbohydrate metabolism, lipid metabolism, amino acid metabolism, cofactors, vitamin metabolism, etc., as well as digestive, endocrine, excretory, and immune systems closely related to insect adaptability (Figure S3).

### 3.3. Correlations between Gut Microbes and FAW Performance

**Figure 5:**
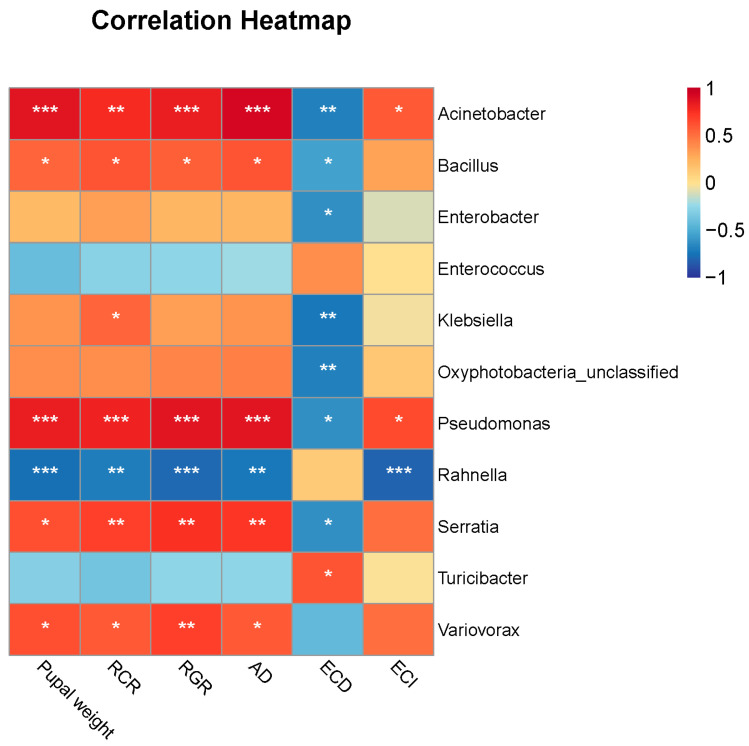
Correlations between biomarkers and FAW performance. Colors showed correlation coefficients. * *p* < 0.05, ** *p* < 0.01, *** *p* < 0.001.

Feeding on different food types changed FAW host adaptation and gut microbiota communities. Therefore, the correlations between gut microbiota and FAW performance were calculated. Among all the biomarkers, the relative abundance of *Enterococcus* in each group was very high. Therefore, we used biomarkers other than *Enterococcus* to analyze the correlation with pupal weight and five nutrient utilization indexes of FAW when feeding on different food types via Spearman’s correlation. The result showed that relative abundances of the genera *Acinetobacter*, *Variovorax*, *Pseudomonas*, *Bacillus*, and *Serratia* were significantly and positively correlated with FAW pupal weight, RCR, RGR, and AD and also demonstrated a significantly negative correlation with ECD (Figure 5). Conversely, the relative abundance of *Rahnella* was significantly and negatively correlated with FAW pupal weight, RCR, RGR, AD, and ECI (Figure 5).

## 4. Discussion

The FAWs’ host range is wide, but their adaptation differs significantly from that of other hosts [6,7]. Herbivore preference and performance can be affected by the varying nutrition profiles and secondary metabolites in plants, which can even induce different levels of insect resistance [43]. In this study, the host fitness of FAWs varied significantly when feeding on four food types. The mean fertility of FAWs was highest when feeding on the artificial diet and maize. When FAWs fed on rice, its reproductive capacity was greatly reduced, resulting in the lowest *r*, *λ*, *R*_0_, and mean fecundity. Compared to maize and wheat, the mean generation time of FAWs fed the artificial diet or rice was significantly extended by about two days. Generally, shorter developmental times and higher reproduction rates on a particular plant species indicate a higher suitability for that plant [44,45,46]. In other words, FAWs exhibited greater adaptability to maize as a host compared to rice which showed the least adaptability. The prolonged generation time of FAWs when feeding on the artificial diet was more likely caused by the poor palatability of the young larvae whose period of development was prolonged on the artificial diet. Compared with the feeding on host plants, FAWs had the lowest RCR when feeding on the artificial diet, confirming that artificial diets are not highly palatable to FAWs. However, this did not affect the high host adaptability of FAWs to the artificial diet, which showed that they had a similarly high fecundity and even higher pupal weight when feeding on the artificial diet than on maize. The results regarding pupal weight and nutrient utilization indexes (RGR, AD, ECI) also indicated that the host adaptation of FAWs followed this descending order: artificial diet > maize > wheat > rice.

Changes in host plants dramatically alter the gut microbiota of herbivores [47]. Proteobacteria and Firmicutes were identified as the most dominant bacterial phyla in *S. frugiperda* collected from four maize-growing regions in Kenya [36]. In this study, the composition and diversity of the gut microbiota of FAWs varied significantly when feeding on different food types, with the highest diversity observed when they were fed maize among the four food types. Besides Firmicutes and Proteobacteria, Cyanobacteria was also identified as the dominant bacterial phylum of FAWs fed on maize. Interestingly, Firmicutes was absolutely dominant in all groups. Especially when FAWs were fed the artificial diet, their gut microorganisms were almost all Firmicutes. This suggests that dietary changes did not affect Firmicutes as the core microbiota, and compositional changes in FAWs’ gut microbiota after dietary alterations were driven by low-abundant bacteria [48,49].

The composition of the gut microbiota significantly influenced insect growth and development [50], with the most dominant contributions of insect gut microbiotas including nutrients and vitamins provision, enhanced digestion efficiency, and the detoxification metabolism of allelochemicals [51,52]. Using PICRUSt2 function prediction, our results also showed that the functions of FAWs’ gut microbiota with significant differences in abundance across the groups are mainly enriched in nutrient (glycan, carbohydrate, lipid, and amino acid) and vitamin metabolisms, as well as the detoxification metabolisms of allelochemicals, etc. Through correlation analysis, five genera (*Acinetobacter*, *Variovorax*, *Pseudomonas*, *Bacillus*, and *Serratia*) and one genus (*Rahnella*) showed significant positive and negative correlations with FAW host fitness, respectively. Beneficial gut microbiotas have been shown to be critical for insect host health [27,53,54]. *Acinetobacter* exhibited enhanced esterase activity and facilitated insecticide metabolism, including cypermethrin and *Bt* toxins, which could contribute to insect resistance [55,56]. *Pseudomonas* may benefit bark beetles by providing nutrients, protecting them from chemical defenses, and antagonizing entomopathogenic fungi [57]. *Serratia* promotes pea aphid development and growth by enhancing fatty acid biosynthesis and metabolism [58]. Although harmful gut bacteria could also adversely affect insects [59,60], the effect of *Rahnella* on insect fitness has not yet been reported.

Based on the differences in host fitness and the gut microbial composition and diversity of FAWs when feeding on different food types, we can make possible inferences about the host adaptation mechanism of the fall armyworm. When the food source is extremely nutritious, such as the artificial diet, the fall armyworm obtains all kinds of nutrients required for growth, development, and reproduction solely through feeding. However, when feeding on host plants, the fall armyworm cannot obtain all the necessary nutrients through feeding alone. Therefore, it is necessary to adjust the community structure of intestinal microorganisms to assist in synthesizing, utilizing, and metabolizing nutrients (such as glycan, carbohydrate, lipid, and amino acids) to promote normal growth and development [61]. When the host plants are nutrient-deficient, and the diversity of the intestinal microbes is insufficient to compensate for the nutrient deficiency caused by food sources, the fall armyworm shows poor performance. The key gut microbiota which affects insect host adaptation and the specific functions of different microbiota strains require further clarification through bacterial clearance and re-inoculation experiments.

## 5. Conclusions

Two-sex life tables and 16s rDNA sequencing analysis were used to determine the host fitness and gut microbial composition and diversity of FAWs when feeding on different food types. Our results showed that the food types significantly affect the host fitness of FAWs. Considering the life history parameters, pupa weight and the nutrient utilization indexes, the fitness of fall armyworms feeding on different food types changed from high to low with the following: artificial diet, maize, wheat, and rice. Similarly, the food types also significantly influenced the gut microbial composition and diversity of FAWs, and those changes were primarily caused by low-abundant bacteria. The functional prediction results for gut microbes with significant abundance differences indicated their main functions involvement in nutrient and vitamin metabolism, as well as other pathways closely associated with host adaptation. Interestingly, we identified five gut genera (*Acinetobacter*, *Variovorax*, *Pseudomonas*, *Bacillus*, and *Serratia*) and one genus (*Rahnella*) that were positively and negatively correlated with the host fitness, respectively. This study revealed the possible role of gut microbes in the host adaptation of FAWs, providing a reference for further exploration into the adaptive mechanisms of FAWs to their hosts and offering a new perspective on pest control.

## Figures and Tables

**Table 1:** Life table parameters of FAWs feeding on different food types.

Parameter	Artificial Diet		Maize		Wheat		Rice	
Intrinsic rate of increase, *r* (d^−1^)	0.19 ± 0.00 a		0.18 ± 0.00 a		0.19 ± 0.00 a		0.15 ± 0.01 b	
Finite rate of increase, *λ* (d^−1^)	1.21 ± 0.00 a		1.20 ± 0.00 a		1.21 ± 0.00 a		1.17 ± 0.01 b	
Net reproductive rate, *R*_0_ (offspring)	573.01 ± 95.19 a		306.99 ± 65.71 bc		321.83 ± 61.13 b		162.15 ± 39.31 c	
Mean generation time, T (d)	32.79 ± 0.28 a		30.79 ± 0.31 b		30.08 ± 0.38 b		32.98 ± 0.69 a	
APOP (for female)	*n*	days	*n*	days	*n*	days	*n*	days
	28	4.03 ± 0.17 ab	20	3.80 ± 0.22 ab	25	3.60 ± 0.21 b	16	4.50 ± 0.35 a
Oviposition days (for female)	*n*	days	*n*	days	*n*	days	*n*	days
	37	6.82 ± 0.22 a	21	6.70 ± 0.46 a	35	7.04 ± 0.67 a	21	7.81 ± 0.59 a
Mean fecundity (for reproductive female)	*n*	offspring/ individual	*n*	Offspring/ individual	*n*	offspring/ individual	*n*	offspring/ individual
	37	1548.68 ± 161.33 a	21	1461.86 ± 134.09 a	35	919.51 ± 122.08 b	21	772.14 ± 114.69 b

Lowercase letters indicate the significance of the difference (compared using the paired bootstrap test, *p*-value < 0.05); *n* represents the sample size.

**Table 2:** The nutrient utilization indexes of FAWs when feeding on different food types.

Indexes	Food Types			
	Artificial Diet	Maize	Wheat	Rice
Dry/fresh weight ratio of food (%)	21.32 ± 0.10 a	8.24 ± 0.08 d	14.19 ± 0.08 c	14.97 ± 0.18 b
Fresh weight of FAW larvae after feeding for 24 h (mg)	322.48 ± 8.64 a	258.55 ± 4.79 b	262.77 ± 7.74 b	221.86 ± 5.86 c
Dry weight of FAW larvae after feeding for 24 h (mg)	58.14 ± 1.97 a	43.80 ± 1.27 b	40.12 ± 1.14 b	24.75 ± 0.60 c
RCR (%)	194.83 ± 5.35 c	381.10 ± 6.58 a	294.33 ± 11.34 b	271.94 ± 5.61 b
RGR (%)	74.13 ± 1.24 a	76.94 ± 1.07 a	56.87 ± 2.64 b	38.25 ± 1.06 c
AD (%)	68.39 ± 1.28 a	45.04 ± 1.16 b	31.87 ± 0.73 c	26.51 ± 1.24 d
ECD (%)	56.87 ± 2.29 a	45.95 ± 2.06 b	61.16 ± 1.99 a	55.66 ± 3.15 a
ECI (%)	38.51 ± 1.08 a	20.29 ± 0.44 b	19.36 ± 0.55 b	14.14 ± 0.43 c

Lowercase letters indicate the significance of difference (compared using ANOVA, followed by Tukey’s HSD post hoc comparisons).

## Data Availability

The data presented in this study are available on request from the corresponding author.

## Supplementary Materials

The following supporting information can be downloaded at: https://www.mdpi.com/article/10.3390/insects15050304/s1. Table S1: Artificial diet formulation for FAWs; Table S2: Statistical table of raw and valid data for 16s rDNA sequencing of gut samples from FAWs larvae feeding on different food types; Figure S1: Venn diagrams of features shared among the guts of FAW larvae feeding on different food types; Figure S2: α-diversity of gut bacteria in FAWs feeding on different food types; Figure S3: PICRUSt2 functional prediction of FAW gut bacteria at KEGG level 2 (*t*-test, *p* < 0.05).
