# Supplementary material for: Gut Microbiota Affects Host Fitness of Fall Armyworm Feeding on Different Food Types
Source: Insects. 2024 Apr 24;15(5):304. doi: 10.3390/insects15050304 (PMC11122019; doi:10.3390/insects15050304)
Supplement: Supplementary file 1 [file insects-15-00304-s001.zip › insects-2960570-supplementary.pdf]

Table S1. Artificial diet formulation for FAWs.

Compositions	Dosage for one serving (g) (mL)*
**Soybean flour**	**80 g**
**Wheat germ flour**	**150 g**
**Yeast powder**	**30 g**
**Sorbic acid**	**3 g**
**Casein**	**40 g**
**Agar**	**20 g**
**Ascorbic acid**	**3 g**
**Multi-Vitamins solution****	**2.5 mL**
**Formaldehyde**	**2 mL**
**Acetic acid**	**4 mL**
**Distilled water**	**1400 mL**

Notes: * The steps for preparing a single artificial diet are as follows: (1) Weigh and mix soybean flour, wheat germ flour, yeast powder, sorbic acid, and casein according to the prescribed dosage. (2) Prepare a solution of ascorbic acid and multivitamins by dissolving them in 100 mL of distilled water according to the prescribed dosage. (3) Weigh AGAR according to the prescribed dosage, pour it into 1300 mL of distilled water, heat until boiling, then turn off the heat. (4) Pour the mixture from step 1 into the boiled AGAR solution, stir well, then add formaldehyde and acetic acid according to the prescribed dosage and stir again. (5) After allowing slight cooling of the mixture from step 4, fully incorporate the mixed solution from step 2 into it by stirring thoroughly. Then pour this mixture into a mold and cool it at 4℃. Depending on requirements, each component's amount can be proportionally scaled up or down to prepare an artificial diet with desired weight.

** The preparation method for the multivitamin solution is as follows: Weigh vitamins B3 (2.75g), B5 (2.75g), B2 (1.38g), B1 (0.69g), B6 (0.69g), BC (0.69g), H (0.55g) and B12 (5.40mg); add distilled water; stir thoroughly to dissolve; and adjust the volume to 100mL. Store at 4 °C for later use.

Table S2. Statistical table of raw and valid data for 16s rDNA sequencing of gut samples from FAWs larvae feeding on different food types.

Sample	Raw_Tags	Raw_Bases	Valid_Tags	Valid_Bases	Valid%	Q20%	Q30%	GC%
AD_1	85112	21.36M	85112	21.36M	100.00	99.19	96.94	54.18
AD_2	83164	20.87M	83164	20.87M	100.00	99.10	96.58	54.18
AD_3	84245	21.15M	84245	21.15M	100.00	99.17	96.80	54.18
AD_4	82580	20.73M	82580	20.73M	100.00	99.17	96.79	54.18
AD_5	83124	20.86M	83124	20.86M	100.00	99.26	97.09	54.18
Maize_1	80553	20.22M	80553	20.22M	100.00	98.64	95.32	54.30
Maize_2	80874	20.30M	80874	20.30M	100.00	98.53	95.05	54.13
Maize_3	84337	21.17M	84337	21.17M	100.00	98.65	95.30	54.64
Maize_4	81882	20.55M	81882	20.55M	100.00	98.58	95.19	54.27
Maize_5	78396	19.68M	78396	19.68M	100.00	98.59	95.22	54.37
Wheat_1	82449	20.69M	82449	20.69M	100.00	99.21	96.96	54.19
Wheat_2	80913	20.31M	80913	20.31M	100.00	99.13	96.73	53.68
Wheat_3	79190	19.88M	79190	19.88M	100.00	99.13	96.57	54.18
Wheat_4	79696	20.00M	79696	20.00M	100.00	99.24	97.06	54.09
Wheat_5	84526	21.22M	84526	21.22M	100.00	99.24	97.09	54.18
Rice_1	80716	20.26M	80716	20.26M	100.00	99.14	96.65	54.21
Rice_2	82310	20.66M	82310	20.66M	100.00	98.75	95.51	54.21
Rice_3	82255	20.65M	82255	20.65M	100.00	97.92	93.47	54.13
Rice_4	82184	20.63M	82184	20.63M	100.00	99.02	96.34	54.22
Rice_5	79114	19.86M	79114	19.86M	100.00	99.22	96.93	54.41

Notes: 'AD' stands for Artificial Diet. For four groups of different food types, each group had 5 samples. Valid%, percentage representation of the ratio between valid data and raw data. Q20%, proportion of data with data quality $\geqslant$ Q20 in the Valid. Q30%, proportion of data with data quality $\geqslant$ Q30 in the Valid. GC%, GC content in the Valid.

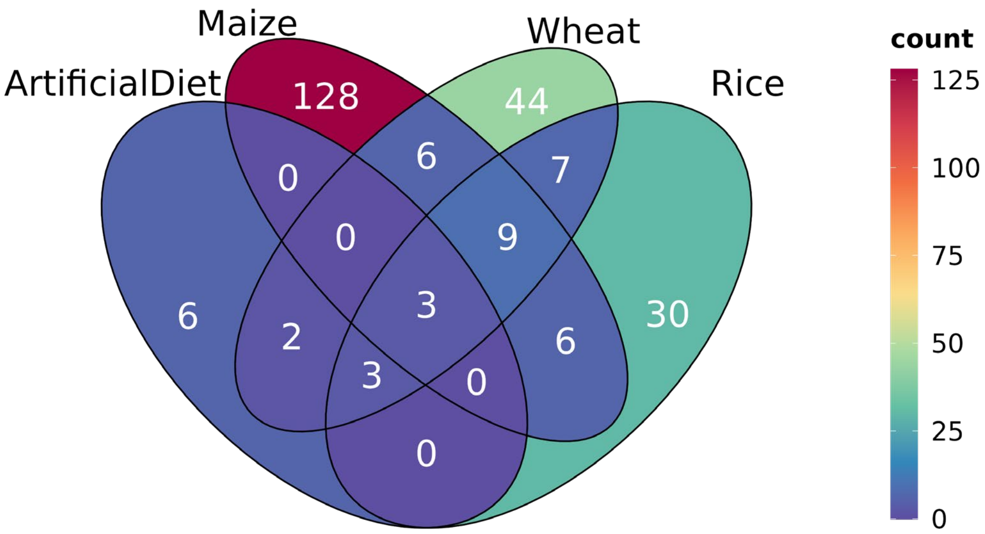

Figure S1. Venn diagrams of features shared among the guts of FAWs larvae feeding on different food types.

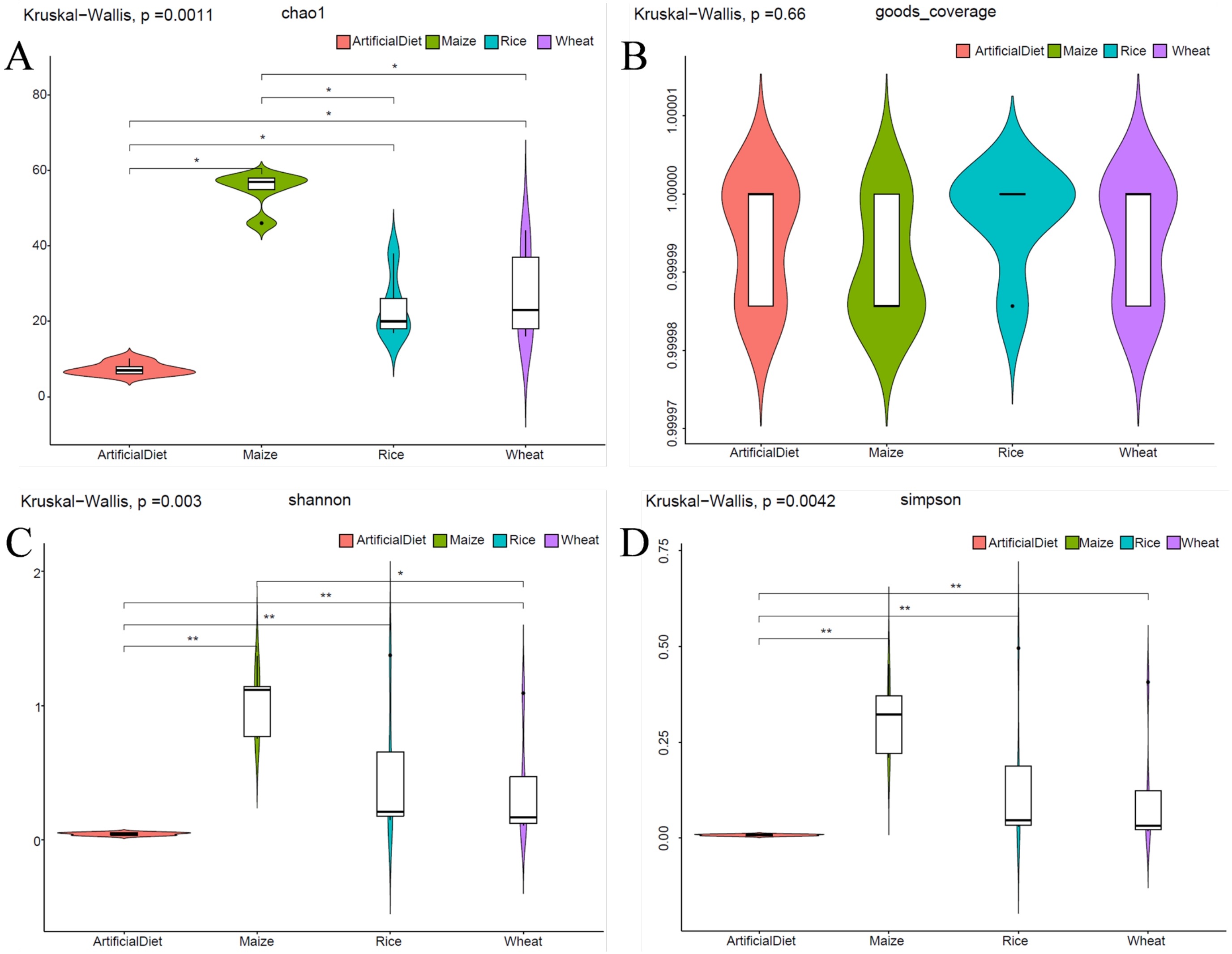

Figure S2. α-diversity of gut bacteria in FAWs feeding on different food types. (A) Significant differences in the Chao index (richness estimator). (B) Significant differences in the Good-coverage index (depth estimator). (C) Significant differences in the Shannon index (diversity estimator). (D) Significant differences in the Simpson index (richness and eveness estimator) (Wilcoxon test; ns: not significant [$P < 0.05$], * $P < 0.05$, ** $P < 0.01$).

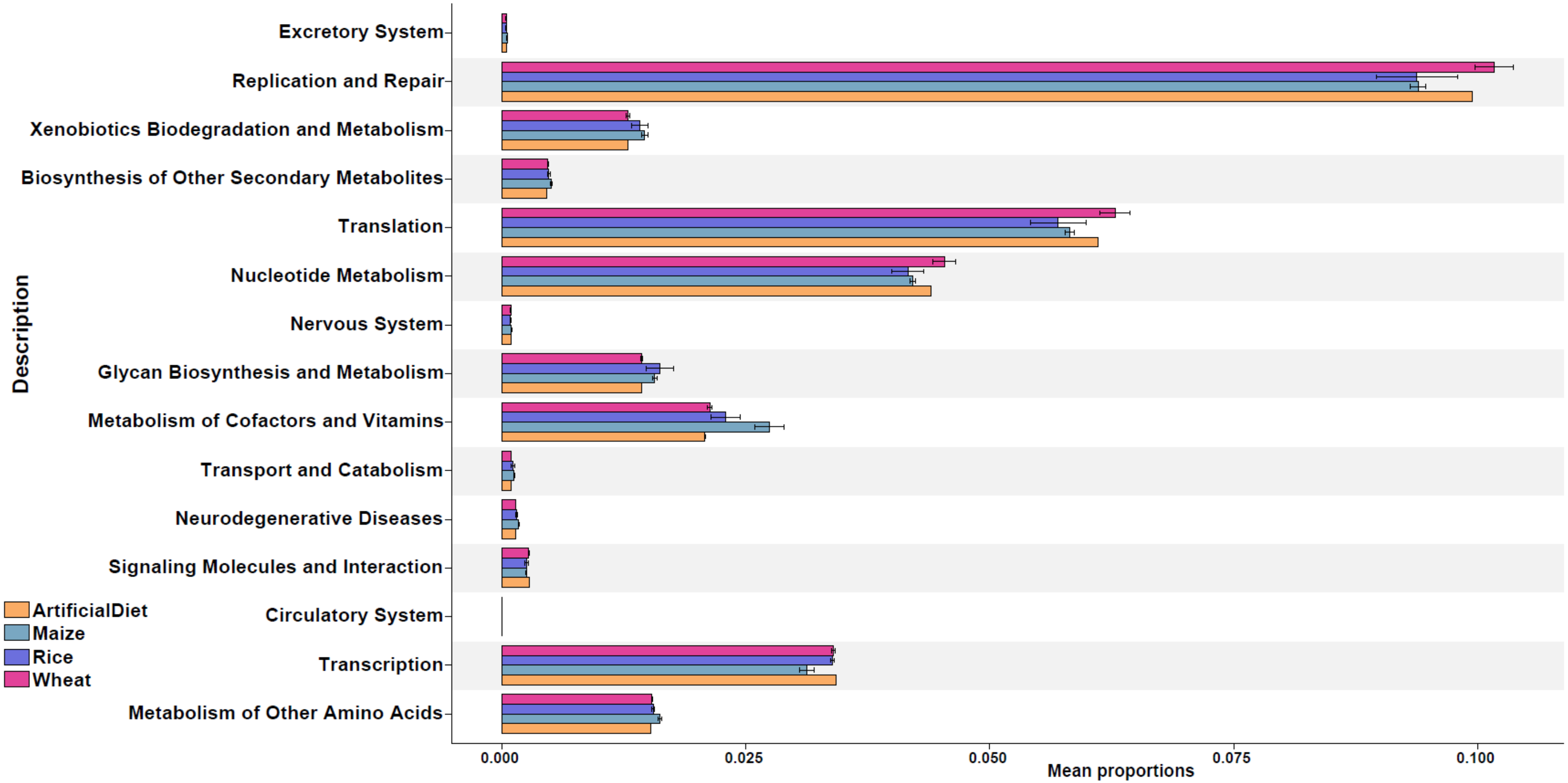

Figure S3. PICRUSt2 functional prediction of FAW gut bacteria at KEGG level2 (t-test, $p < 0.05$).
